# Weather conditions associated with subarachnoid hemorrhage: a multicenter case-crossover study

**DOI:** 10.1186/s12883-021-02312-7

**Published:** 2021-07-19

**Authors:** Michael Kockler, Peter Schlattmann, Mario Walther, Georg Hagemann, Philipp Nils Becker, Steffen Rosahl, Otto W. Witte, Matthias Schwab, Florian Rakers

**Affiliations:** 1 Hans-Berger Department of Neurology, Jena University Hospital, Am Klinikum 1, 07747 Jena, Germany; 2 Institute of Medical Statistics, Computer Sciences and Documentation, Jena University Hospital, 07747 Jena, Germany; 3 Department of Fundamental Sciences, Ernst Abbe University of Applied Sciences, 07745 Jena, Germany; 4 Department of Neurology, HELIOS Hospital Berlin-Buch, 13125 Berlin, Germany; 5 Department of Neurosurgery, HELIOS Hospital Erfurt, 99089 Erfurt, Germany

**Keywords:** Weather, Subarachnoid hemorrhage, Risk factors, Epidemiology

## Abstract

**Background:**

Most spontaneous subarachnoid hemorrhages (SAH) occur unexpectedly and independently of classical risk factors. In the light of increasing climate variability and change, we investigated weather and rapid weather changes as possible short-term risk factors for SAH.

**Methods:**

Seven hundred ninety one patients admitted to three major hospitals in Germany for non-traumatic SAH with a determinable onset of SAH symptoms were included in this hospital-based, case-crossover study. The effects of atmospheric pressure, relative air humidity, and ambient temperature and their 24 h changes on the onset of SAH under temperate climate conditions were estimated.

**Results:**

There was no association between the risk of SAH and 24 h weather changes, mean daily temperature or mean relative air humidity in the overall population. For every 11.5 hPa higher mean daily atmospheric pressure, the risk of SAH increased by 15% (OR 1.15, 95% confidence interval (CI) 1.01–1.30) in the entire study population with a lag time of three days.

**Conclusion:**

Our results suggest no relevant association between 24 h-weather changes or absolute values of ambient temperature and relative humidity and the risk of SAH. The medical significance of the statistically weak increase in SAH risk three days after exposure to high atmospheric pressure is unclear. However, as the occurrence of stable high-pressure systems will increase with global warming and potentially affect SAH risk, we call for confirming studies in different geographical regions to verify our observations.

## Background

The impact of climate change and, thus, the increase in extreme weather events on human health is one of the defining challenges of our time [1]. To build climate resilient health systems, the World Health Organization calls for epidemiological studies to gather knowledge on climate-sensitivity of major diseases and their climate-associated risk factors [2].

One major disease with an incidence of approximately 9 per 100 000 person-years is spontaneous subarachnoid hemorrhage (SAH). The high mortality rate and limited interventional options render SAH a devastating disease: nearly one-third of all patients suffering from SAH do not survive the first month after initial bleeding [3] and nearly half of the survivors remain disabled after another year [4]. Although high blood pressure, current smoking and heavy drinking have long been recognized as general risk factors [5, 6], most SAH occur unexpected and independent of these risk factors. Supported by the observation of a seasonal variation in SAH occurrence [7], it has previously been discussed that certain weather conditions may increase the individual risk of SAH [8–11]. However, the directions of associations that have been observed are conflicting. A recent meta-analysis even failed to pool data on a weather-associated SAH risk because determinants were too heterogeneous [12] leaving the question of an association between weather and risk of SAH unanswered.

The present multicenter study aimed to determine the weather-dependent risk of SAH in a large multicenter study cohort in Germany using the innovative case-crossover study design [13]. The advantage of this model is its self-matching structure that controls for design for individual confounders such as age, sex, socioeconomic status, seasonal influences, chronic disease, and long-term medication. In the light of an increase in extreme weather conditions due to climate change, we also considered rapid changes of weather as a potential risk factor for SAH.

In the current study, we hypothesized that (1) absolute values of ambient air temperature, relative air humidity, and atmospheric pressure, and/or (2) their 24 h changes are associated with the onset of SAH. Determination of a weather associated SAH risk may help to improve population health in an unstable and changing climate.

## Methods

### Study design

For this retrospective and hospital-based case-crossover study, patients were recruited from three secondary or tertiary care hospitals in Central and Eastern Germany: Helios Hospital Berlin-Buch (Berlin), Helios Hospital Erfurt (Thuringia) and Jena University Hospital (Thuringia). The study catchment area exhibits a temperate climate with mild summers and moderately cool winters [14]. Patients’ datasets were retrieved from the respective hospitals data management system and comprised those patients dismissed from the study hospitals with a diagnosis of SAH (I60.x, International Classification of Diseases – 10^th^ Revision). The study extended from January 1, 2003 through to October 15, 2015. Patients over 18 years of age registered as residing in an area of less than 20 km from meteorological stations in the study catchment area were selected. All patient datasets, related patient files including the medical and laboratory reports, emergency records, self and third-party medical history, results of physical examinations, imaging records and original computerized cranial tomographic or magnetic resonance imaging scans were individually screened to identify patients treated for an acute SAH, to verify the diagnosis, to determine the date of SAH onset, and to classify the etiology appropriately [6]. Accordingly, the etiology of each SAH was classified into ‘aneurysmal’ and ‘non-aneurysmal / unknown’ SAH, the latter included those with an unidentifiable cause and patients who died before an angiography could be performed. Diagnosis of SAH was based in most cases on neuroradiological findings consistent with an acute SAH or – if the initial CCT or MRI scan was negative – on the presence of clinical symptoms of SAH and xanthochromic or persistently bloody cerebrospinal fluid [15]. Additional data obtained from patient files included age, sex and aneurysmal size,the World Federation of Neurological Surgeons Grading Score for Subarachnoid Hemorrhage (WFNS) and Fisher grading (SAH graduation based on CT-scan) [16] where applicable.

We excluded patients (1) with inaccessible patient files, (2) that were admitted due to reasons other than acute SAH, (3) with a history of head trauma 96 h before onset of clinical symptoms, (4) with onset of symptoms outside a 20-km radius around the nearest meteorological station, (5) with undeterminable onset of clinical symptoms and (6) when hospital admission was more than three days after onset of clinical symptoms.

Meteorological data for atmospheric pressure, ambient temperature and relative air humidity were provided as hourly values from 19 meteorological stations within the German Weather Service network and averaged over 24 h before statistical analysis.

### Statistical analysis

A case-crossover analysis was conducted to estimate the weather-dependent risk of SAH. This study design was previously developed to study the association between a transient exposure and a potential risk factor for the development of an acute and rare event [13]. As in this elegant variation of the case–control study design, every patient serves as his or her own control, time-invariant factors are controlled for by design, and no additional adjustments are needed (see discussion). In a case-crossover analysis, inference is based on within-subject comparison. We compared the meteorological conditions on different days directly before occurrence of SAH (hazard interval) with meteorological conditions prevailing one week before and one week after the SAH (control interval). Since, in case-only studies, risk estimates are potentially vulnerable to time trends regarding exposure, a bidirectional control interval was chosen to minimize this potential confounder [17, 18].

We analyzed the weather-dependent risk of SAH in two models of multivariable conditional logistic regression (SAS/Version 9.4). Conditional logistic regression was used to take the self-matching structure of the case-crossover design into account. The stratifying variable is the individual patient. The association between atmospheric pressure, ambient temperature and relative air humidity (or their 24 h-changes) and SAH risk is quantified as the odds ratio that describes the change in odds for an event according to alterations in the examined weather variables. In model one, we investigated whether exposure to certain weather conditions affected the risk of SAH. In model two, we determined the effect of the 24 h-changes of weather conditions on the risk of SAH. In both models, the number of days between the hazard interval and onset of SAH is called lag time. Three different lag times, i.e. days one, two and three before onset of SAH symptoms were used in the study to take into account a potential delay before ambient weather conditions fully affect the individual’s risk of SAH. Weather-dependent risk of SAH was determined in the overall study population as well as in the following subgroups: sex (male/female), age (< 60y = young / ≥ 60y = old), and – if available – aneurysmal size (< 7 mm = small / ≥ 7 mm = large). Odds ratios refer to differences in the respective meteorological variable, that represent the third quartile versus the first quartile of all observed values during the study period. As SAH is a relatively rare disease, we interpret odds ratios as relative risks [19].

## Results

### Study cohort

**Fig. 1:**
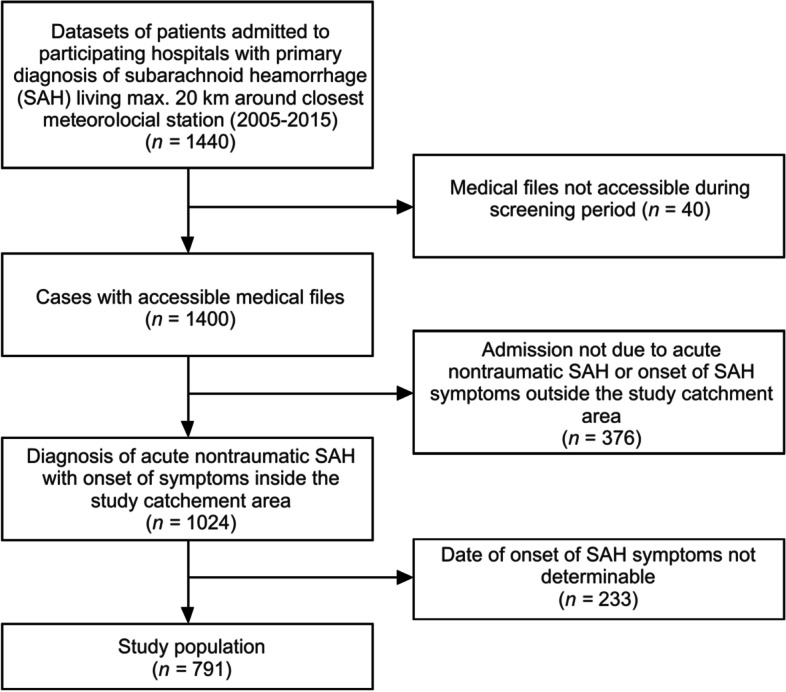
Flowchart of the patient data selection process

Figure 1 shows the study population selection process. 1440 datasets of patients discharged from the three study hospitals with a diagnosis of SAH in the primary position were retrieved from the hospital data management systems. 40 cases could not be processed as the appropriate patient file was not accessible during the screening period. 367 cases were excluded because patients were not admitted for acute SAH (i.e. admission was for control angiography or reimplantation of cranial bone flap), as head trauma was the most likely etiology or as the onset of SAH symptoms was outside the study catchment area. Of the remaining 1024 cases, we excluded a further 233 cases because we were either unable to retrospectively determine the onset of SAH symptoms (i.e. missing data in patient records or intermittent nature of the symptoms) or the time between onset of SAH symptoms and hospital admission comprised more than three days. The remaining 791 patients formed the basis for this study. Patient characteristics are described in Table 1.

**Table 1:** Patient characteristics

	No	%
Overall population	791	
Woman	496	63
Men	295	37
Age		
Young (< 60)	522	66
Old (≥ 60)	269	34
Etiology		
Aneurysmal	568	72
Non-aneurysmal / unknown	223	28
Aneurysm size		
< 7 mm	219	28
≥ 7 mm	209	26
Undetermined	363	46
WFNS		
1–2	494	62
3–5	297	38
Fisher grading		
1–3 (no intracerebral or intraventricular clots)	420	53
4 (intracerebral or intraventricular clots)	371	47

*WFNS* World Federation of Neurological Surgeons Grading System for Subarachnoid Hemorrhage

### Meteorological variables

Meteorological conditions observed in the study catchment area during the study period were typical for a temperate climate with mild summers and moderately cool winters. A summary of distribution of meteorological variables from all participating meteorological stations is shown in Table 2.

**Table 2:** Summarized distribution of meteorological variables over the study period January 1, 2003 through to October 15, 2015

	Minimum	Quartiles			Maximum
		**1**^**st**^	**2**^**nd**^	**3**^**rd**^	
Atmospheric Pressure (hPa)	972	1009	1015	1020	1052
Relative Humidity (%)	26	69	79	87	100
Temperature (°C)	-19	4	10	15	31

### SAH risk in relation to meteorological variables

#### Model 1: Current weather (Fig. 2)

**Fig. 2:**
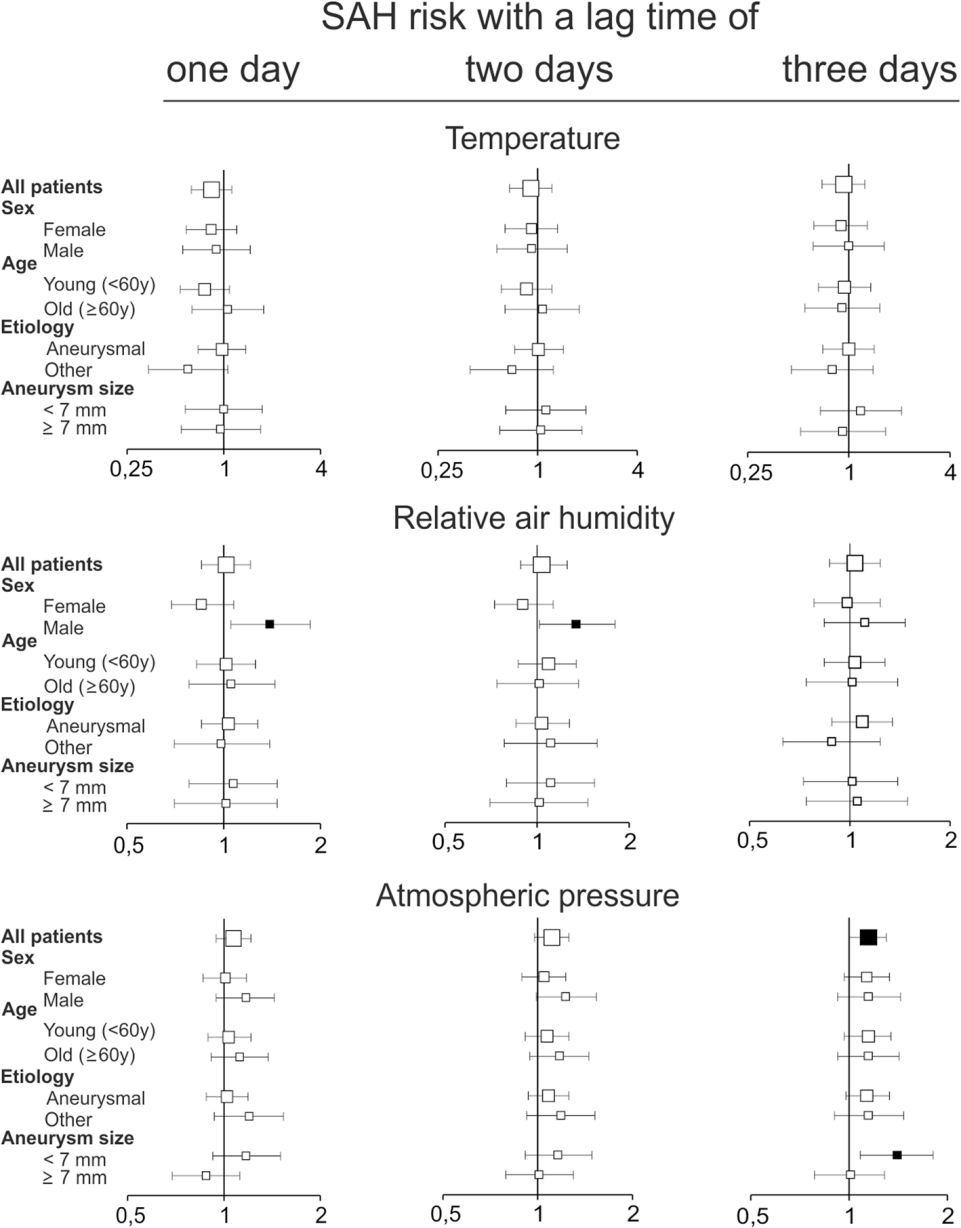
Association of current weather with risk for SAH. Odds ratios for the association of SAH risk and an interquartile range increase in ambient temperature of 11.7 °C, relative air humidity of 17.6% and atmospheric pressure of 11.5 hPa for lag times of 1, 2, and 3 days before onset of SAH symptoms in the overall population and subgroups. Bars indicate 95% confidence interval, filled squares mark odds ratios with confidence intervals not including 1.00

The risk of SAH was not associated with the mean daily temperature in the overall population or subgroups. Similarly, there was no association between the risk of SAH and the relative air humidity in the overall population. However, for an increase in relative air humidity of 17.6%, the risk of SAH increased in males by 39% (OR 1.39, 95% CI 1.05 – 1.86) with a lag time of one day and by 35% (OR 1.35, 95%-CI 1.02 – 1.80) with a lag time of two days. In the overall population, the risk of SAH increased by 15% (OR 1.15, 95% confidence interval (CI) 1.01 – 1.30) for each increase in the mean daily atmospheric pressure of 11.5 hPa, with a lag time of three days (Fig. 2). Similarly, in subgroups, the risk of SAH increased by 41% (OR 1.41, 95%-CI 1.08 – 1.82, Fig. 2) in patients with aneurysms that were smaller than 7 mm.

#### Model 2: 24 h weather changes (Fig. 3)

**Fig. 3:**
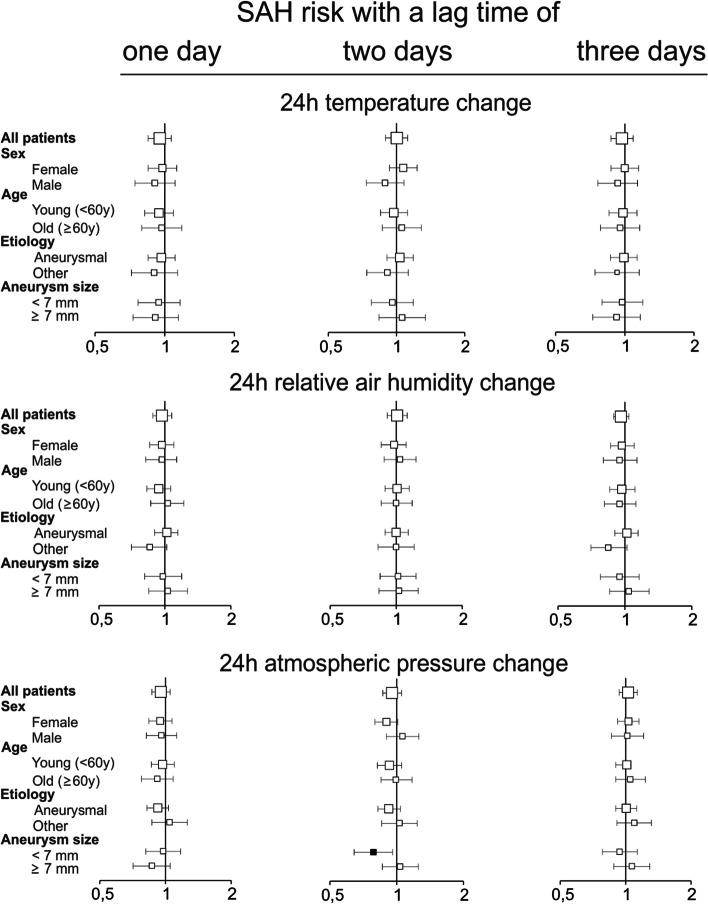
Association of 24 h weather changes with risk for SAH. Odds ratios for the association of SAH risk and an interquartile range 24 h change in ambient temperature of 2.9 °C, relative air humidity of 10.5% and atmospheric pressure of 6.2 hPa for lag times of 1, 2, and 3 days before onset of SAH symptoms in the overall population and subgroups. Bars indicate 95% confidence interval, filled squares mark odds ratios with confidence intervals not including 1.00

There was no association between the risk of SAH and 24 h changes in all three investigated meteorological variables at all lag times except for SAH risk in patients with aneurysms that were smaller than 7 mm. In these patients, risk of SAH increased by 4% (OR 1.04, 95%-CI 1.007 – 1.74, Fig. 3) two days after exposure to a 24 h increase in atmospheric pressure of 6.2 hPa.

## Discussion

This multicenter case-crossover study investigates the risk of spontaneous SAH associated with actual weather or 24 h-weather changes. As the main result, we cannot confirm a relevant association between meteorological conditions and the occurrence of SAH.

We applied the well-established case-crossover design to assess the risk of SAH associated with weather. The case-crossover design is an efficient version of a case–control study design that has been previously developed to investigate the association between a brief and transient exposure and the sudden onset of an acute and rare disease [13]. For example, we and others investigated the relationship between weather and ischemic stroke [20], myocardial infarction [21] and other cardiovascular events [22] using a case-crossover study design. Herein, data were analyzed by means of intra-individual comparison of weather patterns directly before the occurrence of SAH with weather patterns in the same patients´ recent past. Due to this self-matching structure, individual confounders such as sex, age, socioeconomic status, chronic disease, smoking, and long-term medication are controlled for by the study design since every patient acts as his/her own control and these determinants remain constant within individual patients over the sampling period [23]. This is also true for seasonal influences that do not change within the control period. Furthermore, a seven-day control interval allowed matching on a day of the week as a potential confounding temporal factor [23].

Due to the intrinsic nature of weather, patients were exposed to all three meteorological variables at the same time. Moreover, these three meteorological variables are naturally correlated with each other. For example, if the ambient temperature increases, relative humidity decreases and vice versa [24]. A number of previous studies used a univariate approach to assess the influence of weather on the risk of SAH [10, 25–33] and thus, did not consider the physically determined interactions between the meteorological variables. To adjust for these interactions, we conducted a multivariable conditional logistic regression analysis that simultaneously estimates all risk factors in one statistical model.

We also verified all diagnoses and determined the date of symptom onset of the suspected SAH retrospectively and did not solely rely on hospital discharge data. Indeed, of all the cases initially retrieved from the hospitals data management system, we excluded more than 40% of cases because hospital admission had not been due to spontaneous non-traumatic SAH, and either the onset of symptoms was not clearly determinable or the onset was outside the study catchment area. It is likely that study results from previous analyses that regarded hospital admission data as proof of and/or time point of an acute SAH are biased by a similar proportion of patients [8, 32, 34] because meteorological variables and time-point of SAH occurrence are not matched correctly.

Even though we report some statistically significant associations between weather and 24 h-weather changes, these are generally small and most likely not clinically meaningful. Furthermore, we cannot rule out that our statistically significant results are caused by chance due to multiple testing. We decided a-priori to report all statistically significant results in this exploratory epidemiological study and, thus, did not apply additional statistical controlling procedures [35, 36]. In addition, significant results seem to be randomly distributed (Figs. 2 and 3) and do not follow a systematic pattern except for SAH risk associated with atmospheric pressure. Here, exposure to a high atmospheric pressure was associated with an increased SAH risk in the overall population and in patients with aneurysms that were smaller than 7 mm with a relatively long lag time of three days. In the latter group, SAH risk was also associated with 24 h-increases in atmospheric pressure. An elevated SAH risk after exposure to a high or increasing atmospheric pressure as well as after exposure to weather fronts has previously been reported in different geographical regions [28, 37–45]. However, others found an increased SAH risk associated with falling atmospheric pressure [46] or no such association [32, 47, 48] or no association with SAH risk and weather fronts [49]. It is highly likely that especially studies reporting non-existing associations between SAH risk and meteorological conditions are underrepresented because negative results are less frequently published. In this context, it is of interest that one of most comprehensive studies so far conducted in 155 participating hospitals all over the US did not find evidence for a weather-dependent SAH risk [32]. In addition, there is currently no satisfactory physiological explanation for the association between a high atmospheric pressure and SAH, especially if one considers the long lag time of three days. A lag time of three days might indicate an indirect rather than a direct influence of atmospheric pressure on SAH risk, e.g., via the adaptation of human physiological systems to weather or weather changes. For example, it has been speculated that weather dependent changes in blood pressure, serum fibrinogen levels or acute infections may trigger SAH [32]. However, at least in an ambulatory setting, atmospheric pressure is not associated with blood pressure [50]. Furthermore, a relevant association between atmospheric pressure and fibrinogen plasma concentration or infections has not been shown so far.

It is important to note a few limitations of our study. First, we do not have any information on the time spend indoors or outdoors by the individuals during or before the occurrence of SAH. Thus, we cannot really account for the variation in the level of individual exposure to the prevailing weather conditions. However, at least the atmospheric air pressure is not different between indoors and outdoors. Second, even though we only included patients with SAH residing within a radius of less than 20 km from the next meteorological station, the possibility of microclimate influences especially in urban areas cannot be discounted [51]. However, since the vast majority of our patients did not reside in densely developed areas, microclimate influences are most likely negligible. Third, because the meteorological variables are averaged over 24 h, we cannot make a definitive statement about extreme weather conditions that peak within one day. However, these conditions would still be clearly reflected in our averaged values and do most likely not significantly change our results. Finally, our hospital-based study does not include the estimated 6–14% of patients who died of SAH before reaching the hospital, which may have led to an underrepresentation of patients with the most severe bleedings [52]. We acknowledge the possibility that also patients with less severe bleedings are underrepresented as these patients often present with minor symptoms and are misdiagnosed in more than 10% of all cases [53].

## Conclusion

We could not find any relevant increase in the SAH risk associated with the current temperature, relative humidity or their 24-h changes. Increases in atmospheric pressure were weakly associated with an higher SAH-risk, but the significance of this result remains unclear. However, as the occurrence of stable high-pressure systems will increase with global warming and potentially affect SAH risk, we call for confirming studies in different geographical regions to verify our observations and to make reliable policy recommendations for public and health care institutions.

## Data Availability

The data that support the findings of this study are available from the corresponding author upon reasonable request.
